# Stochastic Resetting for Enhanced Sampling

**DOI:** 10.1021/acs.jpclett.2c03055

**Published:** 2022-11-29

**Authors:** Ofir Blumer, Shlomi Reuveni, Barak Hirshberg

**Affiliations:** † School of Chemistry, Tel Aviv University, Tel Aviv6997801, Israel; ‡ The Center for Computational Molecular and Materials Science, Tel Aviv University, Tel Aviv6997801, Israel; § The Center for Physics and Chemistry of Living Systems, Tel Aviv University, Tel Aviv6997801, Israel

## Abstract

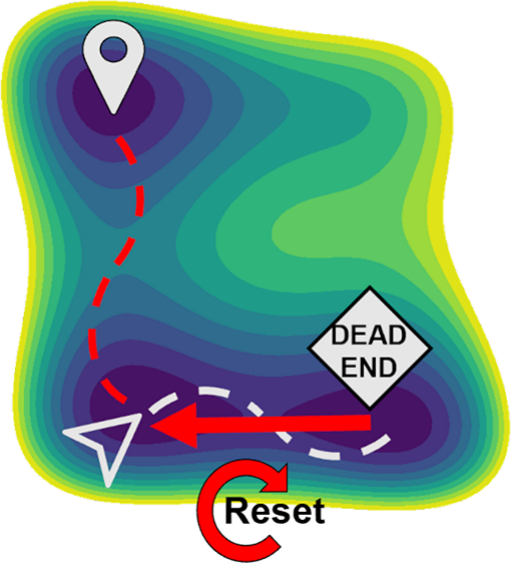

We present a method for enhanced sampling of molecular dynamics simulations using stochastic resetting. Various phenomena, ranging from crystal nucleation to protein folding, occur on time scales that are unreachable in standard simulations. They are often characterized by broad transition time distributions, in which extremely slow events have a non-negligible probability. Stochastic resetting, i.e., restarting simulations at random times, was recently shown to significantly expedite processes that follow such distributions. Here, we employ resetting for enhanced sampling of molecular simulations for the first time. We show that it accelerates long time scale processes by up to an order of magnitude in examples ranging from simple models to a molecular system. Most importantly, we recover the mean transition time without resetting, which is typically too long to be sampled directly, from accelerated simulations at a single restart rate. Stochastic resetting can be used as a standalone method or combined with other sampling algorithms to further accelerate simulations.

Molecular dynamics (MD) simulations are very powerful, providing microscopic insights into the mechanisms underlying physical and chemical condensed phase processes. However, as a result of their atomic spatial and temporal resolution, standard MD simulations are limited to events that occur on time scales shorter than ∼1 μs.^1,2^ In many cases, the complex dynamics of the system lead to longer time scales, through a very broad distribution of transition times between metastable states, also known as first-passage times^3^ (FPTs). To demonstrate this, Figure 1 presents the probability density, denoted by *f*(τ), of the FPTs, τ_1_, τ_2_, ..., τ_*N*_, obtained from *N* simulations of transitions between the two conformers of an alanine dipeptide molecule, a common model system.^3,4^ It shows that many transitions occur on a time scale much shorter than 1 μs, with more than 25% of them under 100 ns. However, the tail of the distribution decays so slowly that the mean FPT is almost an order of magnitude larger, 759 ns, and some trajectories fail to complete even after 4 μs. There is thus an ongoing effort to develop procedures for expediting such processes.^5,6^

**Figure 1:**
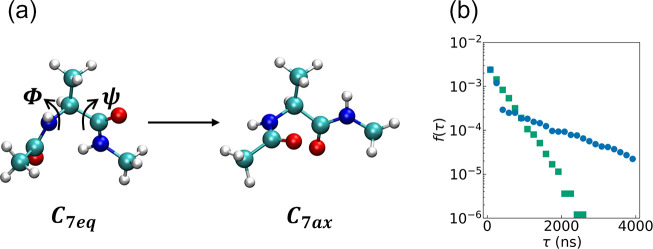
(a) Two conformers of an alanine dipeptide molecule. The white, cyan, blue, and red balls represent hydrogen, carbon, nitrogen, and oxygen atoms, respectively. (b) FPT distributions for transitions between them, starting from *C*_7eq_, without resetting (blue circles) and with Poisson resetting at a rate of *r* = 0.1 ns^–1^ (green squares). The *y* axis is given on a logarithmic scale. The full details of the simulation protocol and how the FPT was determined are given in the Supporting Information.

Stochastic resetting (SR) is the procedure of occasionally stopping and restarting random processes using independent and identically distributed initial conditions. The resetting times are typically taken at constant intervals (“sharp resetting”) or from an exponential distribution with a fixed rate (“Poisson resetting”). The interest in SR has grown significantly since the pioneering work of Evans and Majumdar.^7^ They showed that, while a particle undergoing Brownian motion between two fixed points in space has an infinite mean FPT, its mean FPT with SR becomes finite. Therefore, the particle reaches the target point infinitely faster on average. This result has effectively established an emerging field of research in statistical physics, to which a recent special issue was dedicated.^8,9^

The power of resetting in accelerating random processes has been widely demonstrated in randomized computer algorithms,^10−12^ first-passage and search processes,^13−21^ the convergence of sampling methods, such as Markov chain Monte Carlo and PageRank,^22−25^ queuing systems,^26,27^ experimentally in systems of colloidal particles^28,29^ and in the Michaelis–Menten model of enzymatic catalysis, where resetting occurs naturally by virtue of enzyme–substrate unbinding.^30,31^ The latter finding was then leveraged to develop a general treatment of first-passage processes under restart.^32^ There, it was shown that the FPT distribution in the absence of SR can be used to determine the FPT distribution with resetting. Moreover, the mean and standard deviation of the FPT distribution without resetting are enough to determine a sufficient condition for SR to expedite a random process.^33^ Specifically, if the ratio of the standard deviation to the mean FPT [the coefficient of variation (COV)] is greater than 1, a small reset rate *r* is guaranteed to lower the mean FPT. The slowly decaying distributions that occur in molecular simulations of long time scale processes can also have a COV that is greater than 1. For example, the distribution in Figure 1 has a COV of ∼1.3. This indicates that resetting can expedite MD simulations.

In this work, we use SR for the first time for enhanced sampling of molecular simulations. MD simulations are an exciting playground for the application of resetting while raising new fundamental questions that are of interest to both communities. In SR, the unbiased kinetics (without resetting) are known and the goal is to understand how much speedup can be gained by restarting the random process. On the other hand, in the MD community, the long time scale processes cannot be accessed directly and enhanced sampling methods are required to expedite them. Introducing SR for this purpose raises the question of inference: can we obtain the free energy surfaces and the kinetics of reset-free processes from simulations with SR? This question has not been explored in the SR community but is the natural goal of enhanced sampling methods.

Various methods have been developed in the field of molecular simulations to overcome the long time scale problem, such as umbrella sampling,^34,35^ metadynamics,^1,36−38^ on-the-fly probability enhanced sampling (OPES),^39−41^ and adiabatic free energy dynamics.^42−44^ Many of them rely on identifying suitable collective variables, effective reaction coordinates that ideally describe the slowest modes of the process.^45^ Below, we show that SR can be used for enhanced sampling without finding suitable collective variables, which is highly non-trivial for condensed phase processes.^46,47^ Most importantly, we demonstrate that the mean transition times without resetting, that are often too long to be sampled directly, can be recovered from accelerated simulations performed at a single restart rate. In this Letter, we give a proof of concept for these desirable features using examples ranging from simple models to a molecular system. We obtain a speedup by an order of magnitude in some cases. Our method opens new avenues in both the MD and SR communities, hopefully promoting a fruitful collaboration between the two.

We begin by demonstrating that SR can indeed enhance the sampling of MD simulations. Mathematically, we know that, if the COV is greater than 1, it is guaranteed that resetting can expedite the process. However, for what potential energy surfaces do we expect this to occur? We answer this question using three illustrative model systems representing possible scenarios in MD simulations. Resetting was successful in accelerating transitions in all of them, and for two of them, we obtained an order of magnitude speedup in the mean FPT. To benchmark our approach, we chose the parameters of the model potentials such that the mean FPT without resetting is accessible (∼1 ns) to allow extensive sampling of the unbiased process. Below, we briefly describe the models, while the full parameters are given in the Supporting Information.

The results for each model are given in a separate row in Figure 2. In all cases, the left panel shows the potential and the middle panel presents the FPT probability density *f*(τ) without resetting. The right panel shows the speedup obtained by both Poisson and sharp resetting, at different restart rates *r*. All simulations are of a single particle initialized at fixed positions, denoted by stars in the left panels of Figure 2, with an initial velocity sampled from the Maxwell–Boltzmann distribution at 300 K. We stress that SR is not limited to this choice of initial conditions (see the Supporting Information for other options). The dashed line in Figure 2 defines the spatial threshold for the first passage. The simulations were performed in the Large-scale Atomic/Molecular Massively Parallel Simulator (LAMMPS),^48^ with SR easily implemented in the input files. Full details and input examples are given in the Supporting Information and the corresponding GitHub repository.^49^

**Figure 2:**
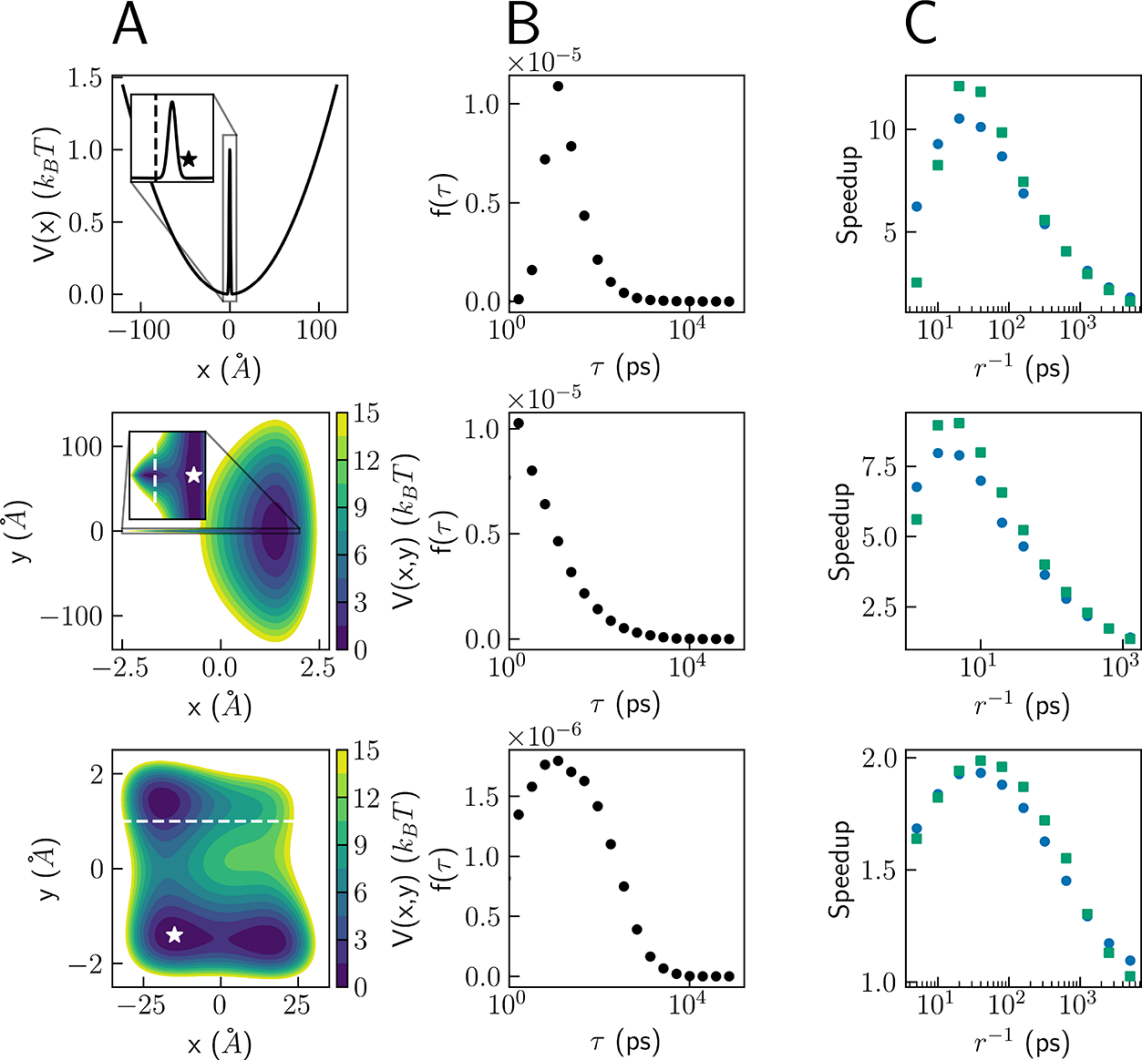
Potential energy surface (column A), FPT distribution without resetting (column B), and speedups obtained using Poisson (blue circles) and sharp (green squares) resetting (column C) for the one-dimensional double-well model (top row), the model of Gimondi et al.^51^ (middle row), and the modified Wolfe–Quapp potential (bottom row). The full potential details are given in the Supporting Information.

The first model is presented in the top row of Figure 2. It is a one-dimensional double-well potential that is composed of a trapping harmonic term and a Gaussian centered at *x* = 0 Å. The model has two symmetric minima that are separated by a moderate barrier (1 *k*_B_*T*). The harmonic spring constant was taken to be soft, such that the particle can explore areas very far away from the center (∼100 Å). This model, with a different choice of parameters, was previously used to describe the umbrella inversion in ammonia.^50^ The simulations were initiated at the right minimum (*x* = 3 Å), and the FPT was defined as reaching the second basin (*x* ≤ −3 Å). The distribution without resetting is broad, spanning about 4 orders of magnitude (note the logarithmic time scale), and has a COV of ∼2.9. In the absence of resetting, some transitions occur as fast as a few picoseconds, while others take as long as tens of nanoseconds. The median FPT is 125 ps, and the mean FPT is 1325 ps. By introducing SR, we were able to reduce the mean FPT by more than an order of magnitude, with a speedup of 10.5 and 12.1 for Poisson and sharp resetting, respectively. The results agree with previous work showing that sharp resetting is guaranteed to lead to higher optimal speedups than any other resetting protocol.^32^

The second model is presented in the middle row of Figure 2. It is a two-dimensional potential, introduced by Gimondi et al.^51^ (with slightly different parameters), to represent two isoenergetic states with very different contributions to the entropy. It has two basins located at (*x* = ±1.3, *y* = 0) Å, which are separated by a barrier of ∼3 *k*_B_*T* centered at the origin. Note that the left basin is so narrow that it can only be clearly seen in the inset of the figure. The basins have the same width in the *x* direction, but in the *y* direction, the right basin is much broader (∼50 Å) than the left basin (∼0.5 Å). As a consequence, the particle can freely explore areas in the right basin where it cannot cross to the other well. The simulations were initiated from the right basin, and the FPT was defined as crossing to the left well (*x* ≤ −1 Å). The results are similar to those of the one-dimensional model. The unbiased FPT distribution is broad, with values ranging from 1 ps to 20 ns. The median and mean of the distribution are 450 and 1125 ps, respectively. The COV is smaller than the COV found for the double-well example (1.44), but the speedup is similar, 8.0 for Poisson resetting and 9.0 for sharp resetting.

The final model system is presented in the bottom row of Figure 2. It is a modified version of the Wolfe–Quapp potential, often used for benchmarking enhanced sampling methods.^45,52,53^ This potential has two metastable basins, one at *y* < 0 and the other at *y* > 0. The former is divided into two substates that have similar width and depth. The lower substates are 30 Å apart and are separated by a moderate barrier (∼1.5 *k*_B_*T*). Larger barriers separate the lower basin from the upper well, ∼6.25 *k*_B_*T* and ∼10 *k*_B_*T* for the left and right lower substates, respectively. This makes the transition to the upper well much more probable from the lower left substate than the right substate. Therefore, this model is an example of a system in which the particle can either cross to the upper well, completing the process, or spend long periods of time in a less reactive nearly isoenergetic state. The simulations were initialized in the lower left substate (*x* = −14.9, *y* = −1.4) Å, and the FPT was defined as crossing to the upper basin, *y* ≥ 1 Å. The obtained FPT distribution without resetting is again very broad, spanning from a few picoseconds for the fastest transitions to tens of nanoseconds for the slowest transitions. We find that, while this model has a very similar COV, mean and median FPT as the second example above (1.43 and 1125 and 500 ps, respectively), the obtained speedup is smaller, ∼2 for both sharp and Poisson resetting. This is because the modified Wolfe–Quapp potential has a mean FPT that is only 2 orders of magnitude larger than the most probable value, as compared to 3 orders of magnitude in the previous example. This result shows that, while a COV greater than 1 guarantees that SR would accelerate the process, the entire shape of the unbiased FPT distribution determines the resulting speedup. In this context, we note a recent development by Starkov and Belan.^54^

It is interesting to test whether SR affects the transition paths between metastable states. We have checked this in Figure 3, plotting trajectories for the modified Wolfe–Quapp potential with transition times representing the mean and median of the FPT distributions with and without resetting. It can be seen that both trajectories with resetting stay localized in the lower left basin before crossing to the upper well, while the trajectories without resetting explore a much broader area of the lower basin, spending more time in non-reactive configurations. The lower panels also show in red the part of the simulations between the last restart and the crossing to the upper well. We find that the final leg of the trajectory shows a similar distribution of transition paths as in the simulations without resetting. This is because SR does not change the dynamics between restart events, unlike other biasing algorithms that continuously add energy to the system,^36,38^ which may result in transitions through highly unlikely paths.

**Figure 3:**
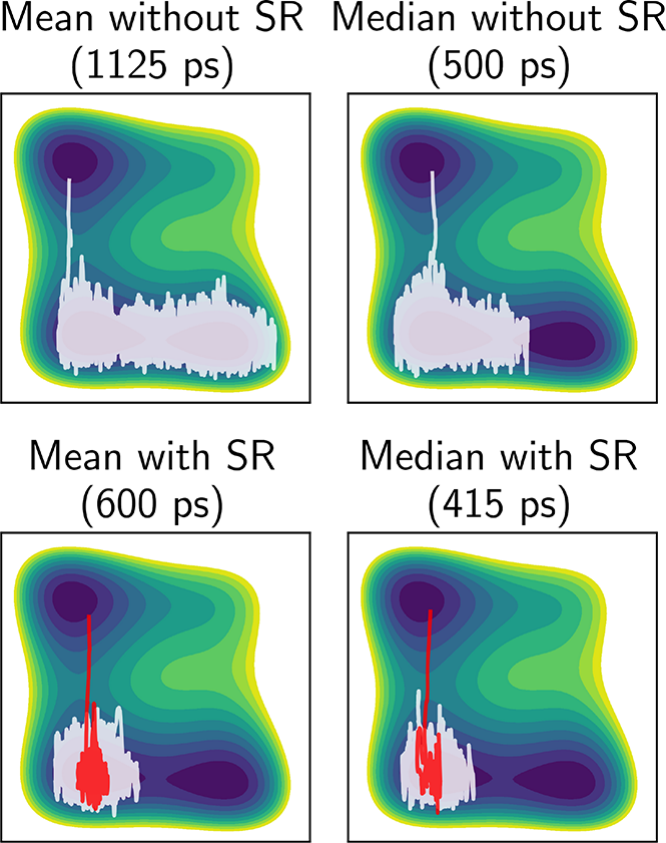
Selected trajectories with FPTs corresponding to the mean and median without resetting (top row) and with sharp resetting every 40 ps (bottom row). The full trajectories are presented in white. For the trajectories with SR, the last leg following the final reset event and until the crossing of the barrier is highlighted in red.

Finally, to demonstrate that SR can be a useful tool in more realistic molecular simulations, we also applied it to accelerate a classic example of enhanced sampling, the alanine dipeptide molecule. It has two states, usually referred to as the *C*_7eq_ and *C*_7ax_ conformers,^36^ which differ by their values of two dihedral angles, ϕ and ψ (see Figure 1a). The simulations were initiated from the more stable *C*_7eq_ conformer after energy minimization, for which ϕ < 0 rad, and the FPT was defined by 0 ≤ ϕ ≤ 2 rad. To the best of our knowledge, this is the first application of SR to a molecular system.

Going beyond the mean FPT, we compare the full distributions with and without resetting in Figure 1b. Our results shed light on how SR leads to acceleration. The distribution without resetting is not exponential and has a COV > 1. Resetting effectively eliminates transition times that are much longer than 1/*r*, leading to a narrower distribution that is very close to exponential, with a COV ≈ 1. A speedup of 2.3 is obtained, reducing the mean FPT from 759 ns without resetting to 333 ns with SR. We find that the speedup is not very sensitive to the resetting rates used from 0.1 to 0.01 ns^–1^ for this system. In such a well-studied model, with known efficient collective variables, methods such as metadynamics or OPES admittedly result in much higher speedups. However, identifying suitable collective variables in condensed phases is still generally very challenging. The great appeal of SR is that no collective variables are needed and only very minimal prior knowledge on the time scales without resetting is required. It is also trivially parallel because different trajectories perform resetting independently of one another. Moreover, SR can be used in a complementary fashion to metadynamics or OPES. These simulations are usually performed with suboptimal collective variables in practice.^45^ If their COV is greater than 1, introducing SR will lead to further speedup.

To conclude the first part of this Letter, our first key finding is that SR is able to expedite transitions in MD simulations ranging from simple models to a molecular system, with up to an order of magnitude reduction of the mean FPT. We examined the sensitivity of the results to the definition of the FPT and the initial conditions (e.g., to sampling the initial position from a distribution). Our findings did not change significantly, and in some cases, the speedups obtained were even greater (see the Supporting Information for a detailed discussion).

Accelerating transitions between metastable states is very useful, because it can be used to generate data for training neural network potential energy surfaces,^55^ to identify collective variables,^46^ and to predict previously undiscovered intermediates.^56^ Next, we tackle another major goal of enhanced sampling: the inference of the unbiased kinetics from biased simulations. Despite many recent advancements,^3,4,53,57,58^ evaluating the rates of long time scale processes from enhanced simulations is still very challenging, and they can deviate by orders of magnitude from experiments.^59^ To increase the accuracy, methods such as infrequent metadynamics or OPES flooding use much weaker biasing,^3,4,53,57^ and the resulting speedups are significantly lower than standard metadynamics. Here, we employ SR for this purpose, showing that it is not limited to expediting transitions but can also be used for inferring kinetics. This is the second key finding of this Letter. Next, we explain how to obtain the mean FPT without resetting using data from accelerated trajectories at a single restart rate.

For long time scale processes (>1 μs), we cannot determine the FPT distribution without resetting. Instead, we can accelerate the simulations and obtain the mean FPT at several reset rates *r* > 0. It is then possible to extrapolate the results to the *r* = 0 limit to obtain an estimate of the unbiased mean FPT. However, this is a very expensive procedure, because typically thousands of transitions are required to converge the FPT distributions and the reset rate that leads to optimal speedup is unknown *a priori*. Fortunately, we find that, for Poisson resetting, the FPT distribution at any reset rate r*, denoted by *f*_r*_(τ), is enough to predict the mean FPT, ⟨τ⟩_*r*_, at all *r* > r* through
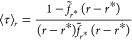
1where the Laplace transform of *f*_r*_(τ) is defined as
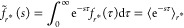
2Equation 1 is exact, given that we have the Laplace transform, and its derivation is given in the Supporting Information. In practice, we evaluate the Laplace transform by performing *N* simulations at a single reset rate r*. We determine *f̃*_r*_(*r* – r*) for a set of discrete values *r* > r* by taking the arithmetic mean of e^–(*r*–r^*^^^)τ_*j*_^, where τ_*j*_ is the FPT of the *j*th trajectory. Then, we use eq 1 to predict the mean FPT for the selected values of *r* > r*. We verify this procedure in Figure 4 for an inverse Gaussian FPT distribution, whose Laplace transform is known analytically. This distribution describes the FPT of drift diffusion to an absorbing boundary.^60^ The full details of the simulations to determine the Laplace transform at reset rate r* numerically are given in the Supporting Information. We find that evaluating the Laplace transform numerically using 10 000 samples is sufficient to accurately reproduce results obtained with the exact transform (see Figure 4a).

**Figure 4:**
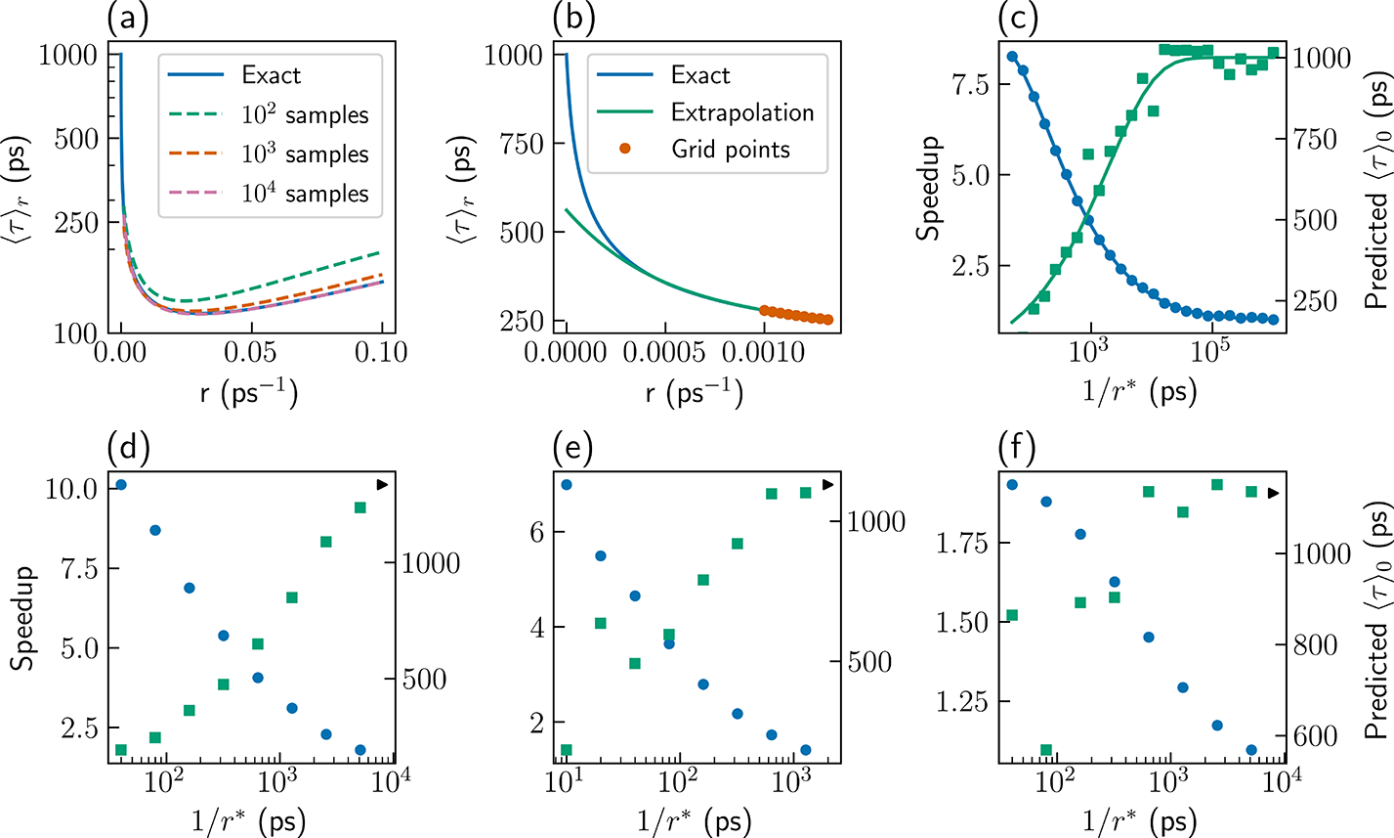
(Top row) Results for an inverse Gaussian distribution with an unbiased mean FPT of 1000 ps (see the Supporting Information for details). (a) Exact ⟨τ⟩_*r*_ obtained using the analytic Laplace transform in eq 1 and approximate values using a different number of trajectories at reset rate r* = 0.001 ps^–1^ to evaluate it numerically. (b) Exact ⟨τ⟩_*r*_ and its fourth-order Taylor series around r* = 0.001 ps^–1^ using the indicated grid points. (c) Speedup (blue circles) and ⟨τ⟩_0_ predictions (green squares), obtained by extrapolation of the Taylor series to *r* = 0, as a function of 1/r*. In panel c, lines represent predictions using the analytical Laplace transform, while the dots show the results using 50 000 trajectories in the evaluation of the numerical Laplace transform. (Bottom row) Speedup (blue circles) and ⟨τ⟩_0_ predictions (green squares) against 1/r* for the (d) one-dimensional double-well potential, (e) potential introduced by Gimondi et al., and (f) modified Wolfe–Quapp potential. The black arrows indicate ⟨τ⟩_0_ obtained in unbiased simulations.

Finally, using the values of ⟨τ⟩_*r*_ predicted from simulations at a single reset rate r* we can extrapolate to *r* = 0 and obtain the unbiased mean FPT at a much lower cost than directly performing simulations at many reset rates. Figure 4b demonstrates the extrapolation procedure. It is based on predicting ⟨τ⟩_*r*_ on a grid of points in the vicinity of r* and fitting them with a fourth-order Taylor series. The mean FPT without resetting is then obtained from the value of the fitted function at *r* = 0. We compared several extrapolation approaches, which resulted in similar accuracy. See the Supporting Information for a full comparison. Figure 4c shows the predicted unbiased mean FPT, ⟨τ⟩_0_, as a function of 1/r*. Naturally, the estimation of the unbiased mean FPT from the extrapolation becomes exact as r* goes to zero. However, the speedup also decreases in this limit. This results in a trade-off between precision and speedup. A similar trade-off was also observed by Ray et al. for the OPES flooding enhanced sampling method.^53^ For this benchmark, we obtained an error of ∼10% in the prediction of the unbiased mean FPT for a speedup of ∼1.7, an error of ∼50% for a speedup of ∼2.8, an error of ∼100% for a speedup of ∼3.9, and an error of ∼500% for a speedup of ∼8.0. Also, in the case of inference, the strength of SR is that it does not require identifying efficient collective variables. While the speedup and accuracy of the kinetic information obtained from other enhanced sampling methods are sensitive to the collective variables used,^53^ resetting has a single parameter, the restart rate, that can be tuned to control the balance between accuracy and speedup.

We have also predicted the unbiased FPT by the same method for the model potentials above. Results are given in panels d–f of Figure 4, as was presented for the inverse Gaussian distribution in panel c. For the one-dimensional model (d), we obtained an error of ∼3% for a speedup of ∼1.7, an error of ∼45% for a speedup of ∼2.8, an error of ∼100% for a speedup of ∼4.1, and an error of ∼595% for a speedup of ∼10.1. Similarly, in the second model system (e), we obtained an error of ∼8% for a speedup of ∼1.8, an error of ∼55% for a speedup of ∼3.1, an error of ∼90% for a speedup of ∼3.6, and an error of ∼515% for a speedup of ∼7.0. For the modified Wolfe–Quapp potential (f), we obtained an error of ∼2% for a speedup of ∼1.4 and an error of ∼30% for a speedup of ∼1.9.

Finally, eq 1 can also be used to find the reset rate, which gives the maximal speedup at almost no cost. This is shown in Figure 4a, in which we tested the sensitivity of the prediction of eq 1 to the number of trajectories used to evaluate the Laplace transform numerically. It can be seen that as little as a hundred samples lead to predictions that capture the qualitative behavior of the mean FPT as a function of the reset rate. While it is insufficient statistics for the inference of unbiased kinetics, it gives a good estimate for the optimal reset rate and speedup.

To conclude, we employed SR to enhance the sampling of long time scale processes in MD simulations for the first time. In applications ranging from toy models to a molecular system, we obtained speedups of up to an order of magnitude in the mean FPT. The most appealing feature of SR as an enhanced sampling method is its incredible simplicity: just restart the simulations at random times to accelerate them. No collective variables are required, and only a coarse estimate of a reset rate that would result in speedup is needed. The optimal speedup can then be predicted through eq 1. We demonstrated the usefulness of SR as a standalone approach to enhance the sampling of MD simulations, but resetting can also be combined with existing algorithms, such as metadynamics, to further accelerate simulations performed with suboptimal collective variables (given a COV > 1). It will be exciting to attempt such a combination on larger and more complex condensed phase systems in the near future.

We also showed that simulations at a single reset rate r* are enough to infer the mean FPT without resetting with adequate accuracy. This is achieved by combining forward prediction to *r* > r*, via eq 1, with backward extrapolation to *r* = 0. In doing so, we have brought inference in SR to the foreground, setting the stage for future theoretical developments. Our method opens new avenues in both the MD and SR communities, hopefully promoting a fruitful collaboration between the two.

## Notes

Example input files are available on the GitHub repository.^49^

## Supporting Information Available

Computational details for MD simulations with and without resetting and sensitivity analyses (PDF)
