# Supplementary material for: Stochastic Resetting for Enhanced Sampling
Source: J Phys Chem Lett. 2022 Nov 29;13(48):11230–6. doi: 10.1021/acs.jpclett.2c03055 (PMC9743203; doi:10.1021/acs.jpclett.2c03055)
Supplement: Supplementary file 1 — jz2c03055_si_001.pdf [file jz2c03055_si_001.pdf]

Supporting Information: Stochastic Resetting for Enhanced Sampling

Ofir Blumer,[†] Shlomi Reuveni,[†,‡,¶] and Barak Hirshberg[*,†,‡]

†*School of Chemistry, Tel Aviv University, Tel Aviv 6997801, Israel.*

‡*The Center for Computational Molecular and Materials Science, Tel Aviv University, Tel Aviv 6997801, Israel.*

¶*The Center for Physics and Chemistry of Living Systems, Tel Aviv University, Tel Aviv 6997801, Israel.*

E-mail: hirshb@tauex.tau.ac.il

General simulation details

All molecular dynamics (MD) simulations were performed in the Large-scale Atomic/Molecular Massively Parallel Simulator (LAMMPS).[S1] The simulations of the one- and two-dimensional models were performed in the canonical (NVT) ensemble at a temperature $T = 300\,K$, using a Langevin thermostat with a friction coefficient $\gamma = 0.01\,fs^{-1}$. The integration time step was $1\,fs$. We simulated a single particle with mass $m = 40\,g\,mol^{-1}$, representing an argon atom. 50000 trajectories were sampled for every model system presented in the main text or in the SI. We checked whether a transition occurred every $1\,ps$ for simulations without resetting, and every $0.1\,ps$ for all simulations with resetting except those with rate $r = 8\,ps^{-1}$, for which we checked every $0.05\,ps$.

The simulations of an isolated alanine dipeptide molecule were performed at $300\,K$ using a time step of $2\,fs$ with a Nosé-Hoover chains thermostat[S2] and a temperature damping

parameter of $100\,\Delta t$. The condition for the FPT was checked every 100 time steps. We used the AMBER99SB force field. Available GROMACS input files for this system[S3] were converted to LAMMPS format using Intermol.[S4] With them, we obtain a free energy difference between the conformers of $\sim\, 9\, kJ\, mol^{-1}$, which is in reasonable agreement with reference values.[S5] The FPT distributions with and without resetting were obtained from 10000 trajectories each. For the trajectories without resetting, 307 trajectories did not show a transition within $4\,\mu s$. They are not plotted in the probability density of Fig. 1 but are included in calculating the mean FPT and speedup. We note that, as a result, the speedup gained by resetting that we report for alanine dipeptide is a lower bound.

Implementation of stochastic resetting

Stochastic resetting (SR) was implemented in the input files as explained below. A stopping mechanism after the first transition was also incorporated through the LAMMPS input. The initial velocities, and their values after each reset event, were sampled from the Maxwell-Boltzmann distribution at the relevant temperatures using Python. For Poisson resetting, waiting times between resets were also sampled using Python, from an exponential distribution, $f(\tau) = re^{-r\tau}$, where r is the restart rate. Below, we give a simplified example of the implementation for a two-dimensional simulation with only three reset events for clarity. The initial position in this example is fixed at $(1, 0)\,\mathring{A}$ and the first transition (passage) is defined as crossing $x = 0\,\mathring{A}$. Full example input files are given in the corresponding GitHub repository.[S6]

```
variable resetTimes index 9 133 22 # Waiting times between resets in ps
variable initialX equal 1
variable initialY equal 0
variable initialVx index -0.00113 0.00278 0.00650
variable initialVy index 0.00120 0.00233 -0.000394
```

```
variable reactionCoordinate equal "x[1]" # The x coordinate of the particle
variable passageCriterion equal 0

label mainLoop
variable a loop 3 # Loop over total number of reset events

label innerLoop
variable b loop ${resetTimes}
run 1000 # Run for 1ps and check whether a transition occurred
if "(${reactionCoordinate} < ${passageCriterion})" then &
   "jump SELF break"
next b
jump SELF innerLoop

set atom 1 x ${initialX} y ${initialX} vx ${initialVx} vy ${initialVy} # Reset...
next a
next Vx
next Vy
next resetTimes
jump SELF mainLoop

label break
print "ALL DONE"
```

Laplace transforms for the inverse Gaussian distribution

We used Eq. 1 for the inverse Gaussian probability density function. The expression for its analytical Laplace transform is given in Eq. 2. We used the parameters $L = 1000$, $V = 1\,ps^{-1}$ and $D = 12500\,ps^{-1}$, which lead to a mean FPT of $1\,ns$ and a coefficient of variation (COV) of 5. In the context of drift diffusion, L is the initial distance from the boundary, V is the drift velocity and D is the diffusion constant.

$$f(\tau) = \frac{L}{\sqrt{4\pi D\tau^3}} \exp\left(-\frac{(L - V\tau)^2}{4D\tau}\right) \tag{1}$$

$$\tilde{f}(s) = \exp\left(\frac{L}{2D} \cdot \left(V - \sqrt{V^2 + 4Ds}\right)\right) \tag{2}$$

To evaluate the Laplace transform of the inverse Gaussian distribution numerically, simulations of first-passage times (FPT) were performed in Python using Scipy[S7] . Trajectories at a reset rate r^* were obtained in the following way: At each step, we sampled a new passage time $\tau_{passage}$ and a new reset time τ_{reset} from their corresponding distributions. If we found $\tau_{passage} < \tau_{reset}$, it meant that there has been a passage before the next reset time. $\tau_{passage}$ was added to the overall simulation time and the simulation was stopped. Otherwise, it meant that the process was restarted before a transition occurred. τ_{reset} was then added to the overall simulation time and we proceeded to sample new values of τ_{reset} and $\tau_{passage}$. We continued this procedure until we encountered a successful passage, $\tau_{passage} < \tau_{reset}$.

Model potentials

Here we present the exact equations and parameters of the chosen model potentials. The parameters are given such that spatial distances are in $\mathring{A}$ and potential energies are in units of $1\,k_BT$ for a temperature of $300\,K$.

The one dimensional double-well is described by Eq. 3, with $A = 1 \times 10^{-4}$, $B = 1$, $C = 1$.

$$V(x) = Ax^2 + B\exp\left(-Cx^2\right) \tag{3}$$

The form of the two dimensional potential introduced by Gimondi et al.[S8] is given in Eq. 4. Most of the parameters are taken as chosen there: $x_1 = 2.5$, $x_2 = -2.5$, $\sigma_1 = 1.3$, $\sigma_2 = 1.3$, $y_1 = 0$, $y_2 = 0$, $\lambda_2 = 1$. To make the right basin broader, we used a larger value of $\lambda_1 = 2000$, and a smaller coefficient for the y-coordinate harmonic spring. In order to achieve an accessible mean FPT in the absence of resetting, we slightly increased the coefficient for the x-coordinate harmonic spring, and lowered the barrier and chose $A_1 = A_2 = 41$.

$$V(x,y) = -\sum_{i=1}^{2} A_i \exp\left(-\frac{(x-x_i)^2}{2\sigma_i^2} - \frac{(y-y_i)^2}{2\lambda_i^2}\right) + 6.7x^2 + 8.4\times10^{-4}y^2 \tag{4}$$

The modified Wolfe-Quapp potential is of the form given in Eq. 5. We modified the original potential[S9] by replacing the x coordinate with a rescaled $x' = x/15$, increasing the coefficients of the linear terms in both coordinates, and multiplying the resulting potential by a factor of 1.5. These modifications were done in order to achieve two remote, distinct sub-states with similar stability in the lower basin.

$$V(x,y) = 1.5\left(x'^4 + y^4 - 2x'^2 - 4y^4 + 1.5x' + 1.2y + x'y\right) \tag{5}$$

Derivation of Eq. 1 of the main text

Here, we will derive Eq. 1 from the main text, which connects the mean FPT at reset rates r, $\langle\tau\rangle_r$, to the FPT distribution at some reset rate r^*, denoted by $f_{r^*}(\tau)$. We begin with Eq. 6, derived by Reuveni,[S10] which connects the FPT distribution without resetting of a random process, $f(\tau)$, to its mean FPT with Poisson resetting at rate r, through

$$\langle\tau\rangle_r = \frac{1-\tilde{f}(r)}{r\tilde{f}(r)}, \tag{6}$$

which is identical to Eq. 1 of the main text for $r^* = 0$. This equation holds for any distribution $f(\tau)$, including the special case $f(\tau) = f_{r^*}(\tau)$. Consequently, we may treat the process with reset rate r^* as if it is an *unbiased* random process and ask what happens when adding a resetting procedure with rate r' to it. We rewrite the equation above for this special case in Eq. 7. The new notation emphasizes that r' is independent of r^* and receives any values $r' \geq 0$, as opposed to r in Eq. 1 of the main text, which only receives $r \geq r^*$. We signify the two independent resetting procedures by two subscripts in the left-hand side of the equation.

$$\langle \tau \rangle_{r^*,r'} = \frac{1 - \tilde{f}_{r^*}(r')}{r' \tilde{f}_{r^*}(r')} \tag{7}$$

What is the distribution of the resulting resetting times? We combined two resetting procedures, each an individual Poisson process with reset times sampled from an exponential distribution, with a rate r^* or r'. Due to the additive property of Poisson processes, this results in another Poisson process, with rate $r = r^* + r'$.[S11] Thus, the combined effect is equivalent to the introduction of a single resetting rate $r = r^* + r'$, meaning $\langle \tau \rangle_{r^*,r'} = \langle \tau \rangle_{r^*+r'} = \langle \tau \rangle_r$. Substituting this fact into Eq. 7 yields Eq. 1 of the main text,

$$\langle \tau \rangle_r = \frac{1 - \tilde{f}_{r^*}(r - r^*)}{(r - r^*)\, \tilde{f}_{r^*}(r - r^*)}. \tag{8}$$

Inference by extrapolation procedure

In this section we will describe in detail the inference procedure used in the main text to obtain the unbiased mean FPT values. We will also present alternative procedures we tested and compare their results. All procedures are based on the FPT distribution with Poisson resetting at reset rate r^*, obtained from simulations.

The chosen procedure (which we denote as method A) begins with predicting the mean FPT at eight equally spaced rates in the vicinity of r^*, $\langle \tau \rangle_{r^*+i\Delta r}$, $i = 1, 2, ..., 8$, which was

done using Eq. 8 (Eq. 1 of the main text). The results given in the main text use a spacing $\Delta r = 0.4r^*$ between adjacent points. Next, the first four derivatives are evaluated at $r = r^*$ using a forward finite difference method, through

$$\left(\frac{d^n \langle \tau \rangle_r}{dr^n}\right)_{r=r^*} = \frac{\sum_{i=0}^{8} C_{n,i} \langle \tau \rangle_{r^*+i\Delta r}}{(\Delta r)^n}, \tag{9}$$

where n is the order of the derivative and $C_{n,i}$ are coefficients given in table S1.[S12] These derivatives are used to obtain an approximate fourth-order Taylor expansion of $\langle \tau \rangle_r$ around $r = r^*$,

$$\langle \tau \rangle_r = \langle \tau \rangle_{r^*} + \sum_{n=1}^{4} \left(\frac{d^n \langle \tau \rangle_r}{dr^n}\right)_{r=r^*} \cdot \frac{(r^* - r)^n}{n!} + \mathcal{O}\left((r^* - r)^5\right). \tag{10}$$

The estimated unbiased mean FPT $\langle \tau \rangle_0$ is simply the value of this function at $r = 0$.

Table S1: Coefficients for the finite difference approximations.

i	0	1	2	3	4	5	6	7	8
$C_{1,i}$	$-\frac{49}{20}$	6	$-\frac{15}{2}$	$\frac{20}{3}$	$-\frac{15}{4}$	$\frac{6}{5}$	$-\frac{1}{6}$	0	0
$C_{2,i}$	$\frac{469}{90}$	$-\frac{223}{10}$	$\frac{879}{2}$	$-\frac{949}{18}$	41	$-\frac{201}{10}$	$\frac{1019}{180}$	$-\frac{7}{10}$	0
$C_{3,i}$	$-\frac{801}{80}$	$\frac{349}{6}$	$-\frac{18353}{120}$	$\frac{2391}{10}$	$-\frac{1457}{6}$	$\frac{4891}{30}$	$-\frac{561}{8}$	$\frac{527}{30}$	$-\frac{469}{240}$
$C_{4,i}$	$\frac{1069}{80}$	$-\frac{1316}{15}$	$\frac{15289}{60}$	$-\frac{2144}{5}$	$\frac{10993}{24}$	$-\frac{4772}{15}$	$\frac{2803}{20}$	$-\frac{536}{15}$	$\frac{967}{240}$

We examined the sensitivity of the extrapolation to the selection of Δr, and found less sensitivity than in other methods. Fig.S1 (a) shows the predicted $\langle \tau \rangle_0$ against $1/r^*$ for different selected values of Δr, for the inverse Gaussian distribution with $\langle \tau \rangle_0 = 1000\,ps$ using the analytical Laplace transform in Eq. 8. It demonstrates that Δr values of different orders of magnitude yield similar predictions.

We also examined fitting directly the mean FPT values at different rates. We will refer to this method as method B. Here, we used a Padé approximant of the form $\langle \tau \rangle_r = \frac{ar^3+br^2+cr+d}{er^2+fr+1}$, which diverges in the limit $r \to \infty$ as does $\langle \tau \rangle_r$. We fitted the function numerically using Scipy[S7] and substituted $r = 0$ to obtain the unbiased mean FPT.

A third method, which we denote as method C, uses a known connection between the mean of a distribution and its Laplace transform, $\langle \tau \rangle = -\left(\frac{d\tilde{f}(s)}{ds}\right)_{s=0}$. We rewrite Eq. 6 as

$\tilde{f}(r) = \frac{1}{1+r\langle\tau\rangle_r}$ and obtain $\tilde{f}(r)$ for several rates $r > r^*$ using $\langle\tau\rangle_{r>r^*}$. Then, we fit numerically a Padé approximant to these selected values of the Laplace tranform. We use the function $\tilde{f}(s) = \frac{a+bs^2+cs}{a+ds^3+es^2+fs}$, which fulfills two general properties of Laplace transforms, $\tilde{f}(0) = 1$ and $\lim_{s\to\infty} \tilde{f}(s) = 0$. Finally, we evaluate the unbiased mean FPT using the derivative of the fitted function at zero, $\langle\tau\rangle_0 = \frac{f-c}{a}$.

Fig. S1 (b) compares between the methods. Though methods B and C do not require equal spacing, here we used eight equally spaced points as needed for method A. Additional tests did not show any better performance for different spacings or number of points. We used $\Delta r = r^*$ since methods B and C proved to be more sensitive to the selection of Δr, and performed well for this value. These methods gave similar predictions to those of method A for most values of r^*, but deviated for others. Since the predictions of method A improved systematically as $r^* \to 0$, as opposed to the predictions of methods B and C, we choose to present this method in the main text.

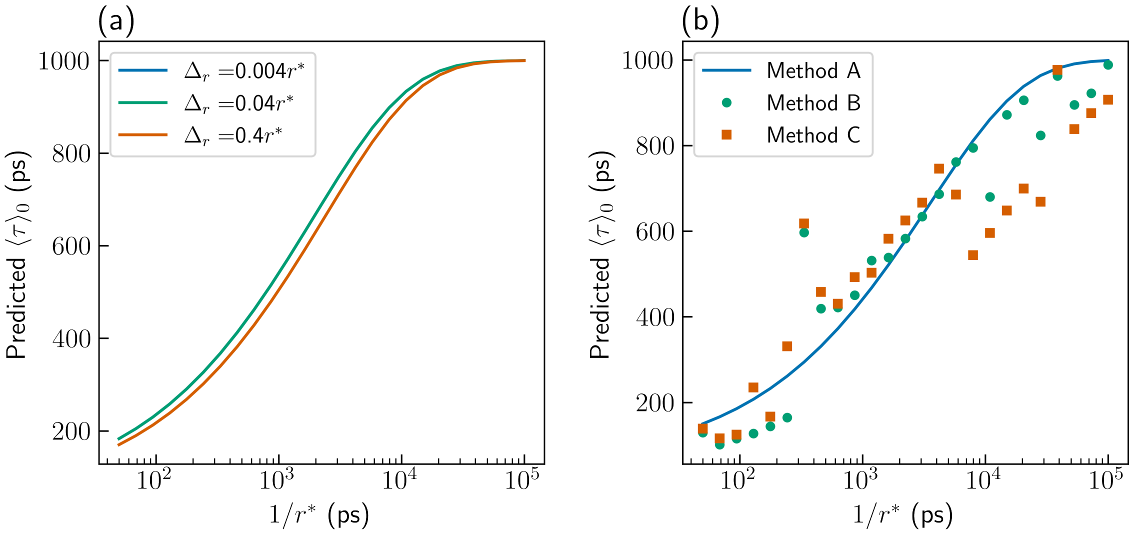

Figure S1: (a) Predictions of $\langle\tau\rangle_{r=0}$ against $1/r^*$ using method A, for different values of Δr. (b) Predictions of $\langle\tau\rangle_{r=0}$ against $1/r^*$ using method A, B and C. The predictions were made using exact values $\langle\tau\rangle_{r^*+i\Delta r}$ for the inverse Gaussian distribution.

Sensitivity to initial conditions and FPT definition

As discussed in the main text, we checked the sensitivity of the method to the definition of the FPT and to the distribution of initial spatial positions. The results presented in the main text are for FPT defined as the first crossing of a fixed value x_1 close to the minimum of the target basin. We performed additional simulations with different values of x_1, and obtained the FPT distributions for these values. The mean and coefficient of variation (COV) of the unbiased simulations in each case are given in table S2, along with the maximum speedups we obtained. The value of x_1 selected for the results presented in the main text is marked in bold. For all model systems, $x_1 = 0$ is the peak of the barrier.

Table S2: Mean and COV of the FPT distributions with no resetting for the three models in the main text, with different values defining the first-passage threshold between states. Also included are the maximum speedups gained for both Poisson and sharp resetting.

**Model**	**Passage value (Å)**	**Mean FPT (ps)**	**COV**	**Speedup**	
				**Poisson**	**Sharp**
One dimensional double-well	0	850	3.58	20.2	18.7
	-1	1050	3.28	16.0	14.5
	-2	1175	3.07	13.9	12.7
	**-3**	1325	2.92	10.5	12.1
	-4	1475	2.82	10.2	9.3
Gimondi et al.	0	875	1.54	9.1	9.9
	-1/3	1075	1.44	8.9	10.1
	-2/3	1125	1.44	8.2	9.4
	**-1**	1125	1.44	8.0	9.0
	-4/3	1225	1.41	6.0	6.9
Modified Wolfe-Quapp	0	1050	1.44	2.0	2.2
	1/3	1100	1.43	1.9	2.0
	2/3	1125	1.43	1.9	1.9
	**1**	1125	1.43	1.9	1.9
	4/3	1125	1.41	1.9	1.9

The results are similar for different choices of x_1. The change in the mean and COV is less than 50% for the one dimensional potential and less than 10% for the modified Wolfe-Quapp potential. The obtained speedups are almost identical for all cases of the modified Wolfe-Quapp potential and very similar for the case of the potential of Gimondi et. al. For the one

dimensional double-well, the speedup was doubled when defining the passage value as the top of the barrier. It should be noted that we only simulated transitions at a single restart rate, that was expected to give the optimal speedup with Poisson resetting according to the unbiased distribution and Eq. 8. It isn't necessarily the optimal rate for sharp resetting, and greater speedups should be expected for sharp resetting when optimizing the restart rate.

The results of the main text used fixed spatial initial conditions. This is equivalent to sampling the positions initially, and after each reset, from a delta function distribution. We examined the influence of the choice of distribution by simulating trajectories with positions sampled from the Boltzmann distribution at the beginning of the simulations and after each reset event. The results are given in table S3.

Table S3: Mean and COV of the FPT distributions with no resetting for the three models in the main text, with initial positions sampled from the Boltzmann distribution. Also included are the expected maximum speedups for both Poisson and sharp resetting.

**Model**	**Mean FPT (ps)**	**COV**	**Speedup**	
			**Poisson**	**Sharp**
One dimensional double-well	6525	1.24	3.5	3.7
Gimondi et al.	1350	1.27	3.3	3.3
Modified Wolfe-Quapp	1750	1.12	1.5	1.5

The mean FPT is greater than the one achieved with fixed initial positions, and the COV is lower. As expected, the speedups are lower as well, because there is a significant probability to initiate the simulations very far from the barrier. Nevertheless, The COV remained greater than one and speedup was obtained using stochastic resetting in all model systems. This verifies that the acceleration gained by SR is not dependent on using a single specific initial condition.

References

(S1) Thompson, A. P.; Aktulga, H. M.; Berger, R.; Bolintineanu, D. S.; Brown, W. M.; Crozier, P. S.; in 't Veld, P. J.; Kohlmeyer, A.; Moore, S. G.; Nguyen, T. D.; Shan, R.;

Stevens, M. J.; Tranchida, J.; Trott, C.; Plimpton, S. J. LAMMPS - A Flexible Simulation Tool for Particle-Based Materials Modeling at the Atomic, Meso, and Continuum Scales. *Comp. Phys. Comm.* **2022**, *271*, 108171.

(S2) Martyna, G. J.; Klein, M. L.; Tuckerman, M. Nosé–Hoover Chains: The Canonical Ensemble via Continuous Dynamics. *J. Chem. Phys.* **1992**, *97*, 2635–2643.

(S3) Bonomi, M.; Bussi, G. PLUMED: Munster Tutorial. 2015; `https://www.plumed.org/doc-v2.7/user-doc/html/munster.html`, Accessed: November 10, 2022.

(S4) Shirts, M. R.; Klein, C.; Swails, J. M.; Yin, J.; Gilson, M. K.; Mobley, D. L.; Case, D. A.; Zhong, E. D. Lessons Learned from Comparing Molecular Dynamics Engines on the SAMPL5 Dataset. *J. Comput. Aided Mol. Des.* **2017**, *31*, 147–161.

(S5) Bonati, L.; Rizzi, V.; Parrinello, M. Data-Driven Collective Variables for Enhanced Sampling. *J.Phys. Chem. Lett.* **2020**, *11*, 2998–3004.

(S6) Blumer, O.; Reuveni, S.; Hirshberg, B. Input Files for 'Stochastic Resetting for Enhanced Sampling'. `https://github.com/OfirBlumer/StochasticResettingForEnhancedSampling`, Accessed: November 10, 2022.

(S7) Virtanen, P.; Gommers, R.; Oliphant, T. E.; Haberland, M.; Reddy, T.; Cournapeau, D.; Burovski, E.; Peterson, P.; Weckesser, W.; Bright, J.; van der Walt, S. J.; Brett, M.; Wilson, J.; Millman, K. J.; Mayorov, N.; Nelson, A. R. J.; Jones, E.; Kern, R.; Larson, E.; Carey, C. J.; Polat, İ.; Feng, Y.; Moore, E. W.; VanderPlas, J.; Laxalde, D.; Perktold, J.; Cimrman, R.; Henriksen, I.; Quintero, E. A.; Harris, C. R.; Archibald, A. M.; Ribeiro, A. H.; Pedregosa, F.; van Mulbregt, P.; SciPy 1.0 Contributors, SciPy 1.0: Fundamental Algorithms for Scientific Computing in Python. *Nat. Methods* **2020**, *17*, 261–272.

(S8) Gimondi, I.; Tribello, G. A.; Salvalaglio, M. Building Maps in Collective Variable Space. *J. Chem. Phys.* **2018**, *149*, 104104.

(S9) Quapp, W. A Growing String Method for the Reaction Pathway Defined by a Newton Trajectory. *J. Chem. Phys.* **2005**, *122*, 174106.

(S10) Reuveni, S. Optimal Stochastic Restart Renders Fluctuations in First Passage Times Universal. *Phys. Rev. Lett.* **2016**, *116*, 170601.

(S11) Kingman, J. F. C. *Poisson Processes*; Oxford Studies in Probability; The Clarendon Press Oxford University Press: New York, 1993.

(S12) Fornberg, B. Generation of Finite Difference Formulas on Arbitrarily Spaced Grids. *Math. Comput.* **1988**, *51*, 699–706.
